# *In silico* approach to reveal viral populations in grapevine cultivar Tannat using transcriptome data

**DOI:** 10.1038/srep15841

**Published:** 2015-10-28

**Authors:** Yeonhwa Jo, Hoseong Choi, Jin Kyong Cho, Ju-Yeon Yoon, Seung-Kook Choi, Won Kyong Cho

**Affiliations:** 1 Department of Agricultural Biotechnology, College of Agriculture and Life Sciences, Seoul National University, Seoul, 151-921, Republic of Korea; 2 Department of Fruit Tree, Korea National College of Agriculture and Fisheries, Jeonju, 560-500, Republic of Korea; 3 Virology Unit, Department of Horticultural Environment, National Institute of Horticultural and Herbal Science, RDA, Wan-Ju, 565-852, Republic of Korea

## Abstract

Viruses are ubiquitous and present in a wide range of settings, from living organisms to various environments. Although viruses are regarded as important pathogens in higher plants, viral populations in specific host plants have not yet been fully examined. This study revealed viral populations in grape berries obtained from a cultivar from a single vineyard using currently available grapevine transcriptomes. Eight viruses and two viroids were identified using 11 grapevine libraries. Virus-associated sequences in each transcriptome ranged from 0.2% (seed) to 8.8% (skin). The amount of viral RNAs and virus copy numbers was quantified, thus revealing the dominant virus or viroid in each individual library. In addition, five viral genomes were successfully assembled *de novo* using transcriptome data. Phylogenetic analyses revealed that the viruses and viroids might have originated from Europe, along with the host. Single nucleotide variation studies revealed the quasispecies of RNA viruses. Taken together, this study defines complex viral populations in three different grape tissues from a single vineyard.

Viruses are ubiquitous and can be found in living organisms, such as bacteria, animals and plants, as well as various environments, such as the sea, volcanoes and air^1^. Plants are hosts for many different DNA and RNA viruses. Due to clonal propagation or grafting, viruses easily infect many horticultural plants compared with seed-propagated plants. In addition, several viruses can simultaneously infect a single horticultural plant. Of the cultivated horticultural plants, grapevines are the most well-known plant host, with more than 64 viruses, including viroids^2^. Infection by a complex of several viruses and viroids in grapevine plants can cause a dramatic reduction of grape production. Researchers have designed several approaches to identify known and novel viruses, such as polymerase chain reaction (PCR) and immunochemical-based methods, which detect a specific known virus^3^. However, these approaches are very time-consuming and too specific, and they cannot reveal the specific combination of viral populations that cause diseases in grapevines^4^.

Recent next-generation sequencing (NGS) technology has enabled the identification of known and novel viruses in infected hosts. For instance, the extraction of virus-enriched RNAs followed by NGS can identify several viruses and viroids infecting grapevines^4^,^5^,^6^,^7^,^8^. However, most studies using NGS have identified only known or novel viruses, and they have not provided further information on viral populations in different tissues and quasispecies of RNA viruses.

Herein, an efficient approach is provided for how to reveal viral populations in different grapevine tissues using available grapevine transcriptomes followed by several bioinformatics analyses.

## Results

### Identification of viruses and viroids from grapevine transcriptomes

**Figure 1:**
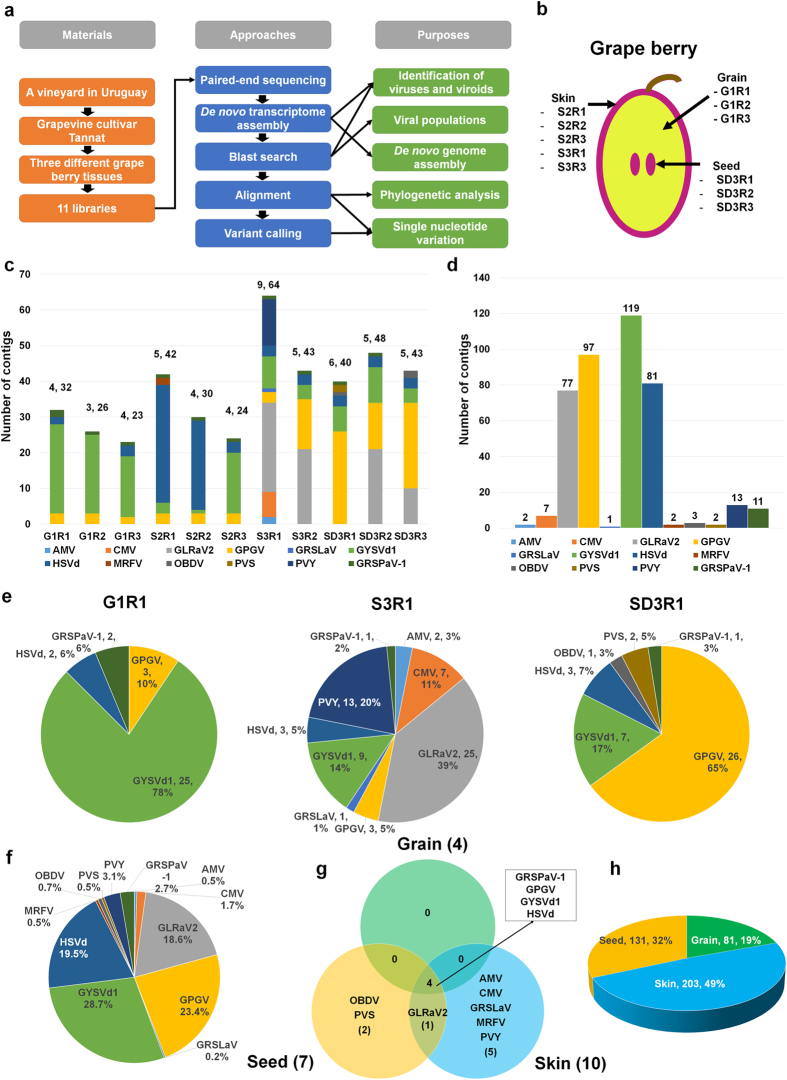
Identification of viruses and viroids from grapevine transcriptomes. (**a**) Data analysis procedures for studying viral populations in grape berries. (**b**) Grape berry illustration showing three different tissues with corresponding libraries. (**c**) Number of contigs assigned to each identified virus. The numbers on the bar indicate the number of identified viruses and total contigs associated with identified viruses in each library. Each identified virus is indicated by a different colour. (**d**) The total number of contigs assigned to each virus identified in the grapevine transcriptome. (**e**) The portion of identified viruses and viroids according to the number of contigs in three representative libraries. The numbers indicate the number of contigs and their percentage. (**f**) The portion of identified viruses and viroids according to the number of contigs from all 11 libraries. (**g**) Venn diagram displaying identified viruses and viroids among the three different tissues (grain, seed and skin). (**h**) The proportion of viral contigs for the three different tissues. The numbers indicate the number of contigs and their percentage.

For the comprehensive study of viral populations through metagenomics, publicly available grapevine transcriptomes were screened to identify the viruses and viroids. Of the screened transcriptome data, a transcriptome of the grapevine cultivar Tannat was used from a previous study, which indicated that the transcriptome contains 119 viral sequences^9^. The transcriptome is composed of 11 libraries from three different grapevine tissues, such as the grain, skin and seed. All transcriptome data were reanalysed for the viral metagenomics study according to the procedures shown in Fig. 1a. In total, five, three and three libraries were constructed from the skin, grain and seed tissues, respectively (Fig. 1b and Supplementary Table 1). To identify the viruses from each grapevine library, *de novo* transcriptome assembly was performed with raw data from each library using the Trinity program^10^. The assembled contigs were blasted against the virus reference genome sequences to identify virus-associated contigs. As a result, 23 to 64 viral sequences were identified from each library (Fig. 1c and Supplementary Table 2). At least three (G1R2 library) to nine (S3R1 library) viruses and viroids were identified in each library. Most of the contigs were obtained from *Grapevine yellow speckle viroid 1* (GYSVd1) (119 contigs) and *Grapevine pinot gris virus* (GPGV) (97 contigs), followed by *Hop stunt viroid* (HSVd) (81 contigs) and *Grapevine leafroll-associated virus 2* (GLRaV2) (77 contigs) (Fig. 1d). Only small numbers of contigs were identified for *Alfalfa mosaic virus* (AMV), *Grapevine rootstock stem lesion-associated virus* (GRSLaV), *Maize rayado fino virus* (MRFV), *Oat blue dwarf virus* (OBDV) and *Potato virus S* (PVS) (Supplementary Table 2). The dominant virus based on the number of virus-associated contigs was different in each library. For example, the contigs associated with GYSVd1 (78%) were dominant in the G1R1 library, while the viral contigs associated with GLRaV2 (39%) were dominant in the S3R1 library (Fig. 1e). GPGV-associated contigs comprised more than half of the SD3R1 library. We combined all the virus-associated contigs and examined the number of contigs assigned to each virus (Fig. 1f). GYSVd1 (28.7%) was the dominant virus, followed by GPGV (23.4%), HSVd (19.5%) and GLRaV2 (18.6%). The identified viruses and viroids were compared among the three different types of tissue (Fig. 1g). Four viruses, including GRSPaV-1, GPGV, GYSVd1 and HSVd, were commonly identified in the three different tissue types (Fig. 1g). Additionally, at least ten different viruses, including two viroids, were identified from the skin tissues, while OBDV and PVS were only identified from the seed tissues (Supplementary Table 2). In total, 203 viral contigs (49%) were obtained from the skin tissues, whereas 81 contigs (19%) were obtained from the grain tissues (Fig. 1h).

### Examination of virus-associated RNAs and their copy numbers

**Figure 2:**
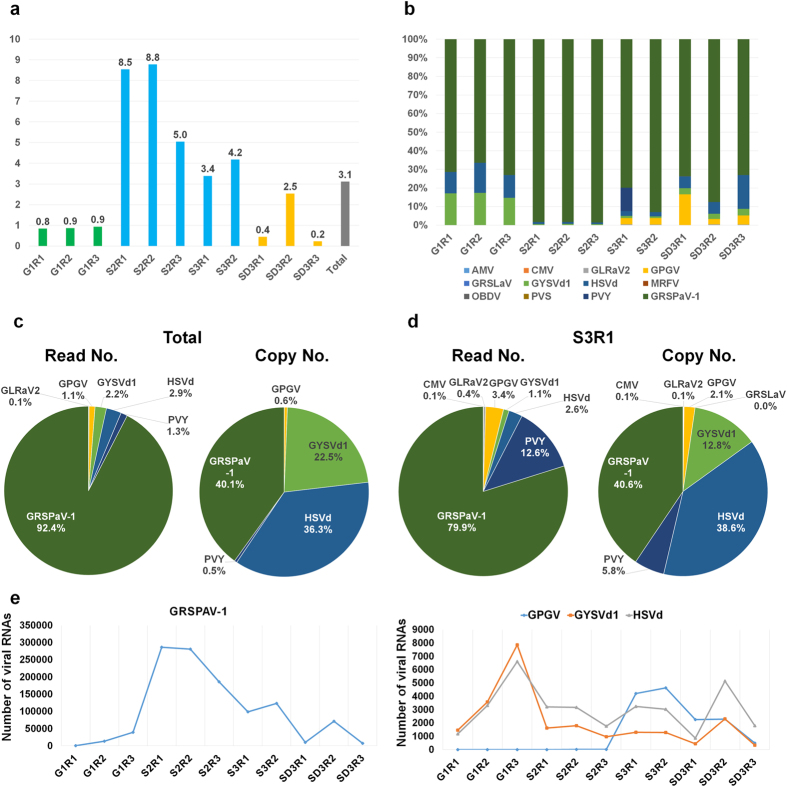
The amount of viral RNAs and the viral RNA copy numbers in the grapevine transcriptome. (**a**) The percentage of viral RNAs calculated based on the number of virus-associated reads divided by the number of total sequenced reads in each sequenced library. (**b**) The relative ratio of individual identified viruses or viroids in each library based on virus-associated reads. (**c**) The percentage of identified viruses and viroids based on virus-associated reads and virus copy numbers in the 11 libraries. (**d**) The percentage of identified viruses based on virus-associated reads and virus copy numbers in the S3R1 library. (**e**) The amount of viral RNAs for GRSPAV-1, GPGV, GYSVd1 and HSVd in each library based on the virus copy number.

Next, raw data was directly blasted against the reference genomes of the identified viruses and viroids. The percentage of virus-associated reads in each library ranged from 0.2% to 8.8% (Fig. 2a). The percentage of virus-associated reads in the three libraries from the grain tissues ranged from 0.8% to 0.9%. Interestingly, there was a high percentage of virus-associated reads in the five libraries from the skin tissues, ranging from 3.4% to 8.8%. The viral reads were the highest in the S2R2 library (8.8%) among the 11 libraries, followed by the S2R1 library (8.5%). The percentage of virus-associated reads in the seed tissues were diverse, ranging from 0.2% to 2.5%. Based on the number of virus-associated reads, we calculated the amount of viral RNAs for each identified virus in each individual library (Fig. 2b). Surprisingly, GRSPaV-1 was the dominant virus in all 11 libraries. Two viroids, GYSVd1 and HSVd, were abundant in the grain tissues. The amount of virus-associated reads in each library seemed to correlate with the size of the individual viral genome. Therefore, the virus copy number was calculated for each virus in each individual library. For example, GRSPaV-1 constitutes 92.4% of all virus-associated sequences based on virus-associated reads; however, when based on virus copy number, this percentage decreased to 40.1%, and the proportion of the two viroids was more than half (Fig. 2c). In the S3R1 library, which contains diverse viruses and viroids, GRSPaV-1 (79.9%) was again dominant, followed by *Potato virus Y* (PVY) (12.8%) based on viral reads (Fig. 2d). However, when based on the virus copy number, the proportion of GRSPaV-1 (40.6%) decreased, while the proportions for HSVd, GYSVd1 and PVY dramatically increased.

Two viruses and two viroids were selected that were commonly identified in all the libraries, and the virus RNA copy numbers were calculated to assess the changes in viral RNAs in each tissue type (Supplementary Table 3). The amount of viral RNAs for GRSPaV-1 was the highest in the skin tissues, including the S2R1 (286,374 copies) and S2R2 libraries (281,343 copies); a small amount of GRSPaV-1 RNA was present in the grain (Fig. 2e). In the seed tissue, including the SD2R2 library, the viral RNAs of GRSPaV-1 (71,627 copies) were abundant. The RNA copy numbers for the two viroids were the highest in the G1R3 library. The amount of HSVd RNAs was higher than that of GYSVd1 in most of the libraries. There was only a small amount of GPGV RNA in the grain tissues and a few of the skin tissues; however, two skin libraries, namely, S3R1 and S3R2, had high copy numbers of GPGV RNA.

### *De novo* genome assembly of viruses and viroids from the transcriptome data

**Figure 3:**
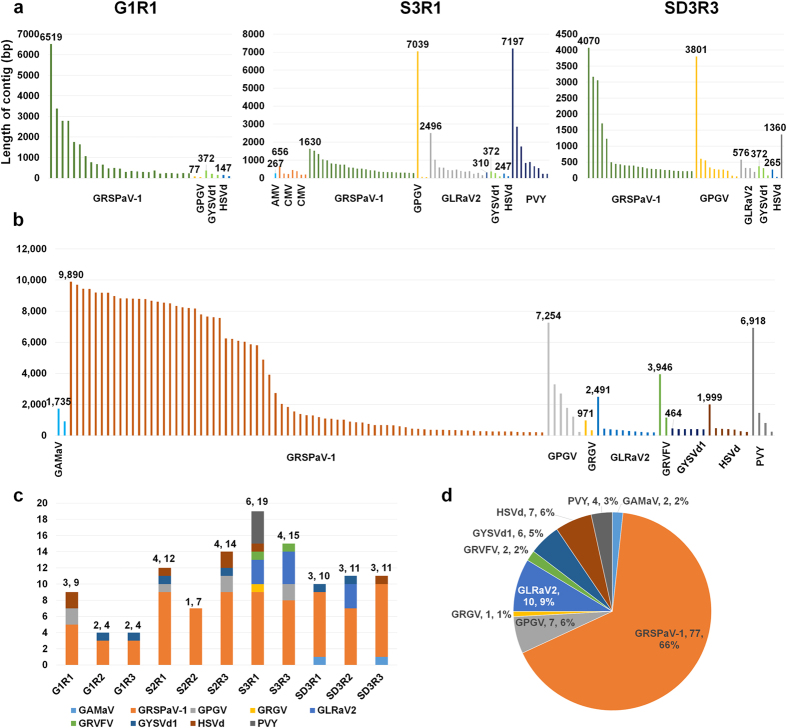
*De novo* transcriptome assembly for obtaining genomes of viruses and viroids. (**a**) The length distribution of viral contigs in three representative libraries, including G1R1, S3R1 and SD3R3. We indicate the length of the longest contig for each virus and viroid. Each virus is depicted by a different-coloured bar. (**b**) The length distribution of the assembled viral contigs, which were de novo assembled using all 11 libraries. (**c**) The number of viral contigs in each library, which were *de novo* assembled using all libraries. The numbers indicate the number of identified viruses and the number of viral contigs. (**d**) The portion of identified viruses and viroids according to the assembled virus-associated contigs from all 11 libraries.

From each library, virus-associated contigs were examined, which were *de novo* assembled (Fig. 3a). For example, 25 contigs were associated with GRSPaV-1 in the G1R1 library; the longest contig was 6,510 bp. In the S3R1 library, a contig of 7,039 bp was the closest to the complete genome of GPGV, and a contig of 7,197 bp was the closest to the complete genome of PVY. From SD3R3, several contigs associated with three viruses and two viroids were identified. Of these, a contig of 372 bp was the closest to the complete genome of GYSVd1. All 11 libraries were prepared from different grape berries in a vineyard. Thus, it was better to combine all the data for the *de novo* assembly of viral genomes. As expected, *de novo* assembly using all 11 libraries generated many virus-associated contigs, which were much longer than those obtained from individual libraries (Fig. 3b). For example, the longest contig associated with GRSPaV-1 was 9,890 bp, which was a nearly complete genome sequence. In addition, of the 77 contigs related to GRSPaV-1, 21 contigs were longer than 8,000 bp. Based on *de novo* transcriptome assembly, we obtained nearly complete genome sequences for GRSPaV-1, GPGV and PVY, and complete genomes for GYSVd1 and HSVd. The five viral genomes were named isolate Tannat. They were consensus sequences of viral populations for each virus in a vineyard. The obtained transcriptome data were again blasted against the virus reference genomes. Three additional viruses were identified, including Grapevine asteroid mosaic-associated virus (GAMaV), Grapevine redglobe virus (GRGV) and Grapevine rupestris vein-feathering virus (GRVFV) (Fig. 3c and Supplementary Table 4). The identified numbers of viruses in each library ranged from one (S2R2) to six (S3R1). The S3R1 library contained the largest number of viral contigs, while the G1R2 and G1R3 libraries possessed four contigs, representing two viruses. According to the viral contigs, which were *de novo* assembled using all 11 libraries, 66% of virus-associated contigs were GRSPaV-1, followed by GLRaV2 (10.9%), HSVd (7.6%) and GYSVd1 (6.5%) (Fig. 3d).

### Phylogenetic analyses of *de novo*-assembled viruses and viroids

**Figure 4:**
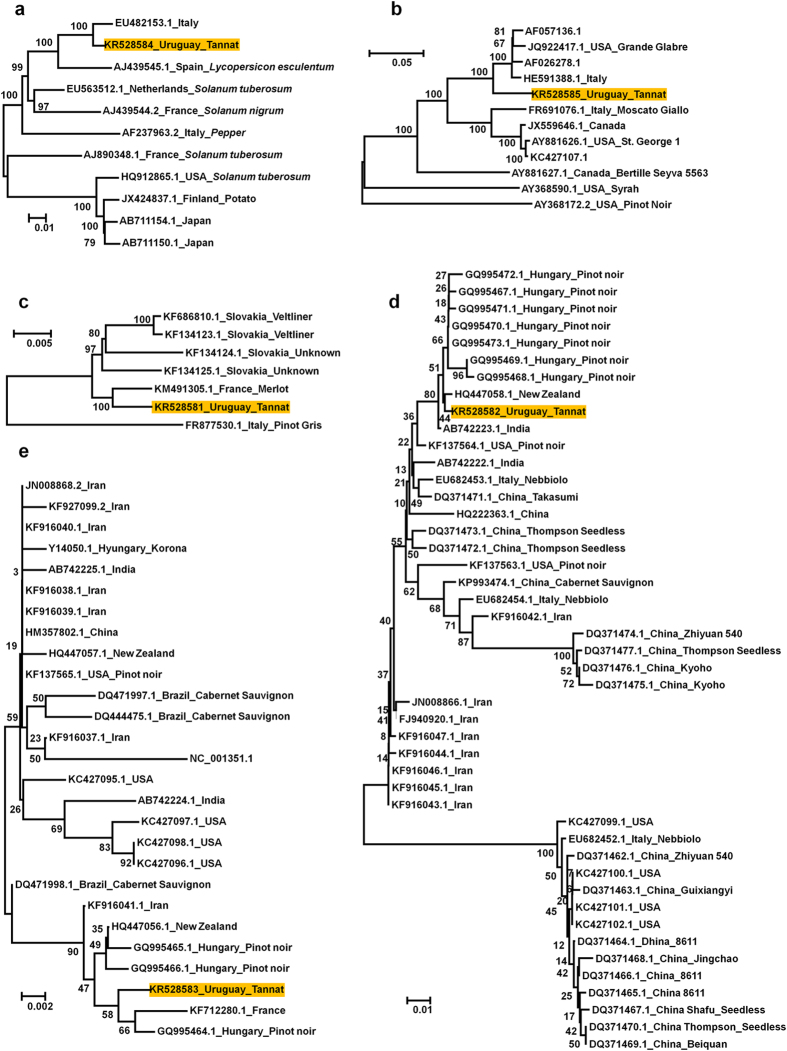
Phylogenetic relationships of three viruses and two viroids. Phylogenetic relationships of PVY (**a**), GRSPaV1 (**b**), GPGV (**c**), GYSVd1 (**d**) and HSVd (**e**). The orange boxes indicate a virus or a viroid with *de novo*-assembled viral genomes in this study, along with their respective accession number. Phylogenetic trees were constructed by the MEGA6 program using the neighbour-joining method with 1,000 bootstrap replicates.

Using the obtained nearly complete genome sequences for three viruses and two viroids, the phylogenetic relationships between the known respective virus and viroid isolates were analysed (Fig. 4). The PVY Tannat isolate was closely related to the PVY isolate from Italy. The GRSPaV-1 Tannat isolate was grouped with four other isolates from the US and Italy. The GRGV Tannat isolate was closely related to the Merlot isolate from France. The phylogenetic tree revealed 46 GYSVd1 isolates, and the Tannat isolate was grouped with many isolates from the pinot noir cultivar from Hungary and an isolate from New Zealand. The phylogenetic tree using 27 HSVd genomes revealed that the Tannat isolate was similar to isolates from France, New Zealand and Hungary.

### Single nucleotide variations of three viruses and viroids in a vineyard

**Figure 5:**
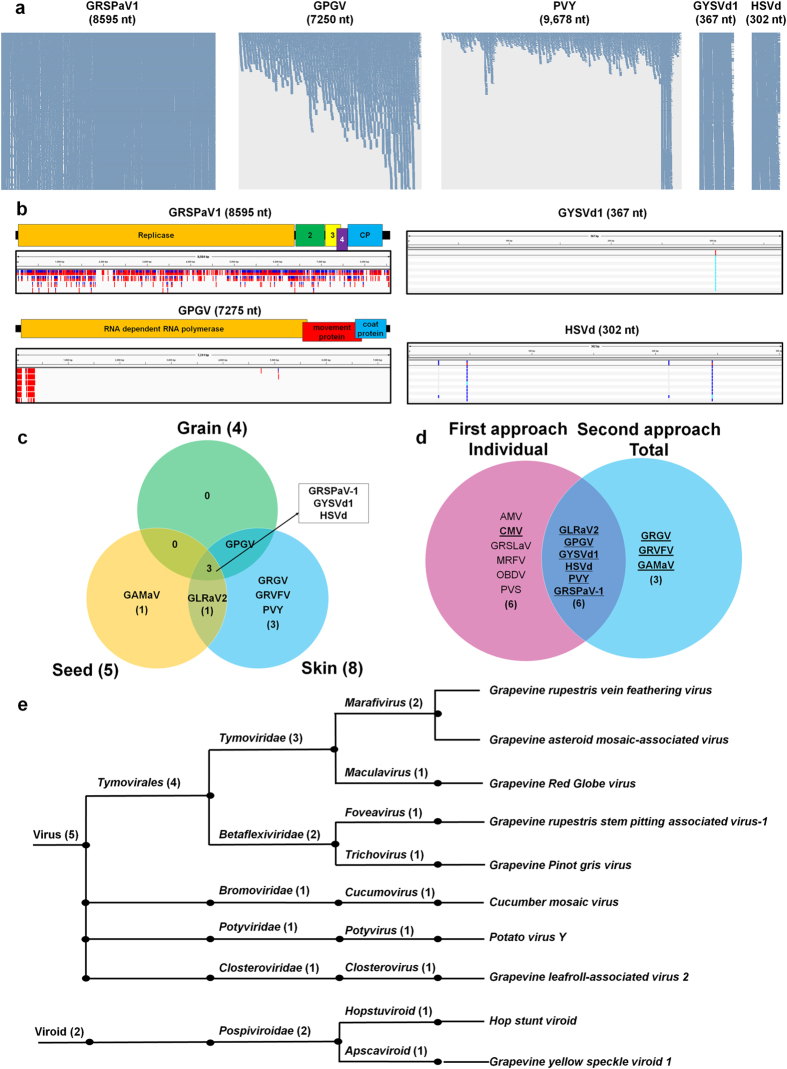
Study of single nucleotide variations and classification of identified viruses and viroids. (**a**) Visualisation of aligned reads on the five viral genomes. (**b**) The various coloured bars indicate the identified SNVs along the genome of each virus or viroid. The number in the coloured box for GRSPaV1 indicates the genes that encode 24.4 kDa, 12.8 kDa and 8.4 kDa proteins. (**c**) A comparison of the viruses and viroids identified from the three different tissues. (**d**) A comparison of the identified viruses and viroids using the two different approaches. The viruses and viroids with underlined, bold characters were then selected. (**e**) Classification of the identified viruses and viroids according to taxonomy.

According to *de novo* viral genome assembly, it seems that several variants for each virus exist in the vineyard. Therefore, the SNVs for five viral genomes were examined. Many virus-associated reads covered nearly whole regions of the GRSPaV1, GYSVd1 and HSVd genomes (Fig. 5a). For GPGV and PVY, the mapped read numbers increased from the 5′ to 3′ regions of the viral genomes. A total of 955 SNVs were identified for GRSPaV1, which were evenly distributed along the genome (Fig. 5b). For GPGV, 200 SNVs were identified, which were specifically identified at the 5′ region of the GPGV genome. From the two viroids, a few SNVs were identified, including one and four for GYSVd1 and HSVd, respectively. No SNVs for PVY were detected.

### Classification of identified viruses and viroids

*De novo* assembly using all the libraries identified seven viruses and two viroids (Fig. 5c). GRSPaV-1, GYSVd1 and HSVd were present in all three tissue types (Fig. 5c). GAMaV1 was only identified in seed tissues, while GRGV, GRVFV and PVY were identified solely from skin tissues. GPGV was identified from both grain and skin tissues, while GLRaV2 was identified from both seed and skin tissues. To identify viruses and viroids from grapevine transcriptomes, two different *de novo* assembly strategies were used. The first was *de novo* assembly using individual libraries, and the second was *de novo* assembly using all 11 libraries together. Ten viruses and two viroids were identified using the first approach, whereas seven viruses and two viroids were identified using the second approach (Fig. 5d). The identified viral contigs were carefully examined manually using BLAST search, and it was concluded that except for the *Cucumber mosaic virus*, most of the contigs that were associated with five viruses from the first approach were not reliable due to the very short sequence lengths. However, the contigs associated with three viruses (GRGV, GRVFV and GAMaV) from the second approach were more reliable due to the relatively long sequence lengths. However, the complete genome sequences for GRGV, GRVFV and GAMaV are not currently available. As a result, eight viruses and two viroids were identified from the grape berries of Tannat grown in a vineyard (Table 1 and Fig. 5e). The identified viruses and viroids belong to one order, six families, nine genera and 10 species. The eight identified viruses belong to five viral families. For example, GRVFV, GAMaV and GRGV are members of the *Tymoviridae* family, whereas GRSPaV-1 and GPGV are members of the *Betaflexiviridae* family. In addition, three viruses were identified belonging to the genera *Cucumovirus*, *Potyvirus* and *Closterovirus*, respectively.

## Discussion

In this study, metagenomics analyses of RNA viruses and viroids were performed using available grapevine transcriptome data. Compared with previous studies associated with plant viral metagenomics^4^,^6^,^7^,^8^, this study is more comprehensive. Viruses and viroids that infect grapevines were identified using NGS techniques, but future directions for assessing viral populations using plant transcriptomes were also provided.

### Seed transmission of two viroids

In the present study, viruses and viroids from different grape tissues were identified and their viral RNAs in each transcriptome corresponding to different developmental stages were quantified. According to the results, grape skin was the most common tissue for the replication of viruses and viroids, followed by seeds and grains. GRSPaV-1, GPGV, GYSVd1 and HSVd were found in the grain and seed tissues. Of them, the seed transmission of GYSVd1 and HSVd has been previously reported^11^. However, it is not known whether GRSPaV-1 and GPGV are also seed-transmitted. The transmission of these two viruses should be studied further.

### Quantification of viral RNAs in the host transcriptome

It was found that the grapevine transcriptomes were enriched with viral RNAs. The most surprising result is that viral RNAs can comprise up to 8.8% of a single transcriptome, such as for the S2R2 library. This result suggests high levels of viral replication in the grapevine host. The authors think that single or several viral pathogens can regulate host transcriptional machinery, thus leading to serious viral disease symptoms. Compared with RT-PCR and protoplast transfection systems^12^,^13^, the present study efficiently quantified viral RNAs within the host transcriptome.

### *De novo* assembly of viral genome from grapevine transcriptome data

There are several approaches for obtaining complete viral genome sequences from infected plant samples. The first is purifying the virus from infected samples, followed by sequencing using NGS or cloning-based Sanger sequencing, such as purifying dsRNAs from infected samples followed by NGS^4^. However, this approach requires an additional step for the virus purification. The second method is small RNA sequencing, which examines virus-derived small interfering (vsi) RNAs, which can be used to find the hot spots of vsiRNAs^5^. The third method is using host transcriptome data^7^,^14^,^15^,^16^. As shown in our study, the enrichment of viral RNAs in the transcriptome enables the assembly of *de novo* viral genomes. Compared with other studies that used NGS data followed by Sanger sequencing, the present study solely used transcriptome data. However, to obtain a nearly complete viral genome, a sufficient amount of viral sequences needs to be acquired. In addition, the obtained genomes were not a single isolate, but a consensus genome sequence of several variants. Furthermore, the phylogenetic analyses using the obtained viral genome provide clues as to where those viruses and viroids might have originated from.

### Single nucleotide variations of viral genomes

RNA viruses and viroids are highly mutated in infected plants, and several variants can often be found in a single plant, which are referred to as quasispecies^17^,^18^. To obtain information on the quasispecies, the SNVs for three viruses and two viroids were examined. No SNVs were identified from PVY, suggesting the presence of a single viral genome. Conversely, many SNVs were identified in the 5′ region of the GPGV genome, suggesting a high level of mutations. The most striking result is that a few SNVs were identified for two viroids. As shown in the authors’ previous study, viroids with hammerhead ribozymes are highly mutated, although the members of the Pospiviroidae family, including GYSVd1 and HSVd, are not severely mutated. It seems that bulk-sequencing targeting a specific viroid is ideal for revealing the quasispecies of viroids in the Pospiviroidae family, as shown in the previous study^19^. We obtained many SNVs along the GRSPaV1 genome, indicating strong quasispecies of GRSPaV1 RNAs, as for other plant RNA viruses^17^.

### The complex of viral populations

The data of this study provide not only a list of viruses, but also assess their possible relationships. For instance, it is interesting that six different virus families can infect a single grapevine cultivar. Of these families, a single virus was not always dominant, thus indicating that different viruses in a host may be competitive or mutualistic^20^. In cases of competition, a single dominant virus or viroid represents the whole viral RNAs, whereas in cases with a mutualistic relationship, viral disease symptoms may be worse due to the synergistic effects of the viruses. In addition, it is important to examine viral populations based on the viral copy number rather than viral RNAs. As shown in the present study, the amount of viral reads from GRSPaV-1 was overwhelming; however, according to the viral copy number, two viroids comprised more than half of the total viral populations.

### Application of viral transcriptomics for studying viral ecology

Prior to the generation of virus-free seedlings, it was necessary to obtain a list of viruses that infect specific grapevine cultivars. Therefore, a list of viruses and viroids that infect a grapevine cultivar is helpful. One of the interesting outcomes from this study is that the changes in virus copy number can be monitored according to plant developmental stages and in different tissues. For instance, the amount of viral RNA for GRSPAV-1 was the highest in the skin in stage 2, but gradually decreased in the skin in stage 3. Of course, many samples and replicates are necessary to confirm these findings. The examination of viral populations in different cultivars within the same orchard, during different seasons and in different orchards could provide further helpful data. So far, many plant virologists have focused on a single virus in a single host; however, as shown in this study, viruses can form viral complexes, which can regulate host transcriptome machinery. Thus, the authors believe that viral ecology associated with viral communities and populations, along with environmental changes, will be the future direction of research on plant viruses^21^. Taken together, this study reports the successful application of plant transcriptome data using bioinformatics analyses to reveal the complexes of viral communities in grape berries.

## Methods

### Plant materials and library preparation

Information on the plant materials and library preparation were described in detail in a previous study^9^. In brief, grape berries were sampled at three different growth stages, including 1, 5 and 7 weeks post-flowering, from the grapevine cultivar Tannat, which was grown in a vineyard of 2,000 plants in Melilla, Montevideo, southern Uruguay during the 2011 to 2012 growing season. Three independent pools of 30 berries were sampled for each developmental stage. However, the authors do not know whether the sampled materials were obtained from symptomatic or non-symptomatic plants. Total RNAs were extracted using the Spectrum Plant Total RNA kit (Sigma-Aldrich, Carlsbad, CA, USA) according to the manufacturer’s instructions. The RNA libraries were prepared using the TruSeq RNA Sample Prep Kit (Illumina, San Diego, CA, USA). Sequencing was performed using a HiSeq 1000 sequencer generating 101-bp paired-end reads.

### *De novo* transcriptome assembly

All bioinformatics analyses were performed with a workstation (two six-core CPUs and 256-GB RAM) operated using the Ubuntu 12.04.5 LTS operation system. Two paired-end sequenced FASTQ files for each library were first subjected to *de novo* transcriptome assembly using the Trinity program (version 2.0.2, released 22^nd^ January 2015) with default parameters^10^. Eleven grapevine transcriptomes from the individual libraries and a whole transcriptome were obtained by combining all the libraries. In addition, the Velvet/Oases assembler was used to assemble the whole grapevine transcriptome^22^,^23^. For the *de novo* assembled transcriptome using the Velvet/Oases assembler, a CD-HIT program with a threshold of 90% was applied to filter redundant contigs^24^.

### Identification of viruses and viroids

To identify the viruses and viroids that infect grapevine berries, three different approaches were used. For the first, *de novo* assembled contigs were blasted against complete reference sequences of viruses and viroids (http://www.ncbi.nlm.nih.gov/genome/viruses/). For the BLAST search, the MEGABLAST algorithm with a cut-off e-value of 1e-5 was applied. MEGABLAST is much faster than other sequence similarity programs and provides very reliable sequence similarities for the identification of viruses and viroids. For the second approach, the same BLAST search was performed against complete reference sequences of viruses and viroids using a whole grapevine transcriptome, which was assembled using the 11 libraries. For the third approach, the raw data from each library were directly blasted. For this, all the FASTQ files were converted into FASTA files using the FASTX-Toolkit (http://hannonlab.cshl.edu/fastx_toolkit/).

### *De novo* genome assembly of three viruses and two viroids

To obtain complete or nearly complete genome sequences for the viruses and viroids, the whole grapevine transcriptome was first blasted against complete reference sequences of viruses and viroids. Among the contigs with high levels of similarity, the longest contig for each virus or viroid was selected. *De novo* assembled viral genome sequences were manually compared with the known reference viral genome sequences. Finally, nearly complete genome sequences for three viruses were obtained, including GRSPaV-1 (accession number KR528585), GPGV (accession number KR528581) and PVY (accession number KR528584), as well as complete genomes for two viroids, including GYSVd1 (accession number KR528582) and HSVd (accession number KR528583).

### Single nucleotide variations

To identify single nucleotide variations (SNVs), all the raw data were aligned on the obtained genome sequences for three viruses, including GRSPaV-1, GPGV and PVY, and two viroids, including GYSVd1 and HSVd, using the Burrows–Wheeler Aligner (BWA) with default parameters^25^. The obtained SAM files were converted to BAM files for sorting using SAMtools^26^. The BAM files were sorted for SNV calling using SAMtools. Afterward, mpileup was conducted to generate the VCF format. The SNVs were then called using BCFtools implemented in SAMtools, and finally, SNVs were filtered using BCFtools. In addition, the MAQ program was used to identify SNVs using easyrun script^27^. For the visualisation of mapped reads on the genome, aligned SAM files were imported to the Tablet program^28^.

### Phylogenetic analyses

For phylogenetic analyses, phylogenetic trees were constructed for GRSPaV-1, GPGV, PVY, GYSVd1 and HSVd using the MEGA6 program^29^. In brief, nucleotide sequences for the *de novo*-assembled genome and the known genome of the corresponding virus or viroid were aligned by the ClustalW program implemented in MEGA6. A large number of genomes were available for PVY; to reduce the number of PYV genomes for phylogenetic tree construction, ten PVY genomes that were highly matched to the isolate Tannat were used. There were many complete genomes for the GYSVd1 and HSVd isolates. Therefore, the genomes of only two viroids were selected, which were identified from the grapevine. Aligned nucleotide sequences were used to construct a phylogenetic tree using the neighbour-joining method with 1,000 bootstrap replicates.

## Additional Information

**How to cite this article**: Jo, Y. *et al.* *In silico* approach to reveal viral populations in grapevine cultivar Tannat using transcriptome data. *Sci. Rep.* **5**, 15841; doi: 10.1038/srep15841 (2015).

## Supplementary Material

Supplementary Information

Supplementary Dataset

## Figures and Tables

**Table 1:** Summary of identified viruses and viroids infecting grapevine berries.

Index	Abbreviated name	Reference	Size	Total read no.	Total copy no.	Genome assembly	SNV	Grain	Skin	Seed
1	GRVFV	AY706994.1	6,617	89	1.36	NA	NA		P	
2	GAMaV	AJ249357.2	1,852	329	17.9	NA	NA			P
3	GRGV	AF521977.1	2,006	224	11.2	NA	NA		P	
4	GRSPaV-1	NC_001948.1	8,744	1,139,276	13159.5	8,595	955	P	P	P
5	GPGV	NC_015782.1	7,275	13,948	193.6	7,250	200	P	P	
6	CMV	NC_002034.1	3,357	63	1.9	NA	NA		P	
7	PVY	NC_001616.1	9,704	15,533	161.7	9,678	0		P	
8	GLRaV2	NC_007448.1	16,494	1311	8.0	NA	NA		P	P
9	HSVd	NC_001351.1	302	35,659	11925.7	302	4	P	P	P
10	GYSVd1	NC_001920.1	366	26,804	7396.7	367	1	P	P	P

The table contains detailed information on the viruses and viroids identified in our study. Complete genome sequences are not currently available for GRVFV, GAMaV and GRGV. NA indicates not available. SNV represents the number of single nucleotide variations. P indicates the presence of a virus or viroid. The number in the genome assembly column indicates the sizes of the *de novo* assembled viral genomes.
