# Supplementary material for: In silico approach to reveal viral populations in grapevine cultivar Tannat using transcriptome data
Source: Sci Rep. 2015 Oct 28;5:15841. doi: 10.1038/srep15841 (PMC4623741; doi:10.1038/srep15841)
Supplement: Supplementary Information [file srep15841-s1.pdf]

Supplementary Information for

In silico approach to reveal viral populations in grapevine cultivar Tannat using transcriptome data

Yeonhwa Jo[1], Hoseong Choi[1], Jin Kyong Cho[2], Ju-Yeon Yoon[3], Seung-Kook Choi[3], and Won Kyong Cho[1*]

[1]Department of Agricultural Biotechnology, College of Agriculture and Life Sciences, Seoul National University, Seoul, 151-921, Republic of Korea

[2]Department of Fruit Tree, Korea National College of Agriculture and Fisheries, Jeonju, 560-500, Republic of Korea

[3]Virology Unit, Department of Horticultural Environment, National Institute of Horticultural and Herbal Science, RDA, Wan-Ju, 565-852, Republic of Korea

[*] Corresponding author

Dr. Won Kyong Cho

Department of Agricultural Biotechnology, College of Agriculture and Life Sciences, Seoul National University, Seoul, 151-921, Republic of Korea

Tel: +82-2-880-4687; Fax: +82-2-873-2317

E-mail: wonkyong@gmail.com

Supplementary Tables

Supplementary Table 1.

Summary of 11 grapevine libraries used for the identification of viruses and viroids.

Supplementary Table 2.

Summary of identified viruses and viroids from *de novo*-assembled contigs using individual libraries.

Supplementary Table 3.

Summary of identified viruses and viroids from *de novo*-assembled contigs using all 11 libraries.

Supplementary Table 4.

Summary of identified virus-associated contigs, which were *de novo*-assembled using all 11 libraries.
